# COVID-19 among slaughterhouse workers: relationship between worker’s health and food safety

**DOI:** 10.47626/1679-4435-2022-794.

**Published:** 2022-03-30

**Authors:** Simone Domingues Garcia, Manoela de Carvalho, Mariângela Baltazar

**Affiliations:** 1 Enfermagem, Universidade Estadual do Oeste do Paraná (Unioeste), Cascavel, PR, Brazil.; 2 Odontologia, Unioeste, Cascavel, PR, Brazil.

**Keywords:** occupational health, coronavirus infections, public policy, health personnel, food safety

## Abstract

**Introduction:**

COVID-19 pandemic has impacted the entire society, as well as different work processes. The food industry remained operational since the first cases were reported in Brazil, and activities were only interrupted by municipal health surveillance agencies when COVID-19 cases were identified among workers. This has raised the debate about worker’s health and sanitary measures in the work environment.

**Objectives:**

To analyze the number of COVID-19 cases between March and September of 2020 among slaughterhouse workers in the state of Paraná and its correlation with food security policies.

**Methods:**

This was a quantitative, exploratory, and descriptive study that analyzed secondary data from the Health Department of the state of Paraná, Brazil.

**Results:**

A significant number of COVID-19 cases were reported among slaughterhouse workers in Paraná. The importance of workers’ health and food security should be discussed, including aspects such as food production, quality, and access. The workers included in this study represent an important workforce that influences food supply to different regions and countries. Considering that the virus is highly contagious and has impacted the lives of local workers and entire communities, the role of the government and collective actions should be emphasized.

**Conclusions:**

The monitoring of health surveillance agencies and new cases should be conducted as a strategy to promote prevention and care during the pandemic.

## Introduction

In Brazil, Federal Law no. 11,346 of 2006 created the National Food and Nutrition Security System (Sistema Nacional de Segurança Alimentar e Nutricional, SISAN) and defined the concept of food and nutritional security as the guarantee of everyone’s right to regular and permanent access to quality and sufficient food without compromising the access to other essential needs. However, COVID-19 pandemic exacerbated the challenges related to the guarantee of this right to families and populations.^1^

The food issue and its variables have always been the subject of discussions not only at the national level, but also at the global level. Regarding food security, Brazil occupies a prominent position in relation to other countries because of its imposing influence on food production, as well as on the establishment of agribusiness, which has important repercussions on the economic, social, and political sectors. The economic sector plays a more significant role in food security given its active participation in aspects such as the supply of products to meet domestic demands, employment of a significant portion of the workforce, and major involvement in exportation.^2^

Despite external factors such as the COVID-19 pandemic, the food production industry follows the same logic of every other production process, whose purpose is to accumulate and not to guarantee people’s right to food and nutrition. Food is produced by a few transnational companies that control the entire process of food production, distribution, and consumption, thus limiting people’s options.^3^

The COVID-19 pandemic revealed the current food production and distribution system to be involved in the beginning of the pandemic and its subsequent spread around the world. The possible relationship between agribusiness and the pandemic is little disclosed, especially regarding the current model of agri-food production, particularly of extensive livestock raising. This model includes aspects such as the confinement of large herds and the environmental impact caused by the destruction of large ecosystems, which could originate new viruses and bacteria that could cause human diseases, as well as the advantages of food production in slaughterhouses, which were identified as places of significant contamination and virus transmission in the regions where they are located.^4,5^

Importantly, since the first cases of COVID-19 were reported in Brazil, the food industry has remained operational. Only a few slaughterhouses in the South region had to stop production after being interdicted by municipal health surveillance agencies^5^ due to the number of cases and working conditions that could increase virus transmission. Although the Brazilian food production sector is an essential service, it was proven to be flawed and vulnerable: on the one hand, food supply is not sustainable, given the number of workers that were infected in the workplace; on the other hand, many small family farmers were unable to sell their products due to restrictions that prevented fairs, schools, and local businesses from opening, which negatively impacted both the income and the access to food of the working class.^6^

However, when we consider the number of workers involved in the vast chain of food production and distribution, including rural workers, factory workers (such as in slaughterhouses), and those who work in food transportation, sourcing, supply, and distribution, there is a significant portion of people exposed to the virus who receive little attention. Understanding viral dynamics and controlling the spread of COVID-19 are still challenging for scientists and health managers around the world. In addition to social and spatial characteristics that account for an urban-regional mode of transmission, the role of production sectors, particularly those considered to be essential services (such as slaughterhouses), as places of increased COVID-19 transmission in the regions where they are located has also been investigated.^5^

This study was therefore designed to analyze the number of confirmed cases of COVID-19 among slaughterhouse workers in the state of Paraná, Brazil, and its correlation with food security policies.

## Methods

We conducted a quantitative study that analyzed secondary data from the State Worker’s Health Center (Centro Estadual de Saúde do Trabalhor, CEST) of the Health Department of the state of Paraná (Secretaria de Saúde do Paraná, SESA) and from the Epidemiological Bulletin published by SESA.

Quantitative studies obtain results through investigations in which numerical information is gathered and subsequently presented in boxes, tables, and measurements. Quantitative studies deal with numbers and use statistical models to explain the data.^7^

The data used in this study are publicly available and can be accessed through online government information systems. Because only publicly available data were used, the study was not evaluated by a research ethics committee, as recommended by Resolution no. 466/2012 of the Brazilian National Health Council.

The time frame included the beginning of the pandemic in March 2020 until data tabulation in September 2020.

Data were analyzed using simple statistics. The number of confirmed cases of COVID-19 among slaughterhouse workers was correlated with the total number of cases reported in Paraná until September 2020. Study variables included confirmed cases of COVID-19 among the general population and confirmed cases of COVID-19 among slaughterhouse workers.

Inferential statistics were performed using the public domain software BIOSTAT®. Independence levels were calculated using the chi-square test; α = 5% indicated the presence of correlation between study variables.

## Results

The state of Paraná comprises 399 municipalities that are administratively divided by the State Health Department into four macroregions, which are subdivided into 22 health regions. Data were presented according to each health region and the number of workers in each slaughterhouse; the total number of slaughterhouses in Paraná and cases notified to health services were considered (Table 1).

**Table 1:** Distribution of confirmed cases of COVID-19 among slaughterhouse workers according to each health region in the state of Paraná, Brazil, March to September 2020

Macroregions	HR	General population	Confirmed cases of COVID-19 among the general population	Number of slaughterhouse workers	Confirmed cases of COVID-19 among slaughterhouse workers	Prevalence of COVID-19 in the general population	Prevalence of COVID-19 in slaughterhouse workers	PR between the general population and slaughterhouse workers	Chi-square test for K proportions (p-value)
East	2	3,615,027	68,857	1,684	60	1.90	3.56	1.87	< 0.0001
	3	631,810	6,417	3,849	28	1.02	0.73	0.72	0.0924
	5	455,880	2,036	223	10	0.45	4.48	10.04	< 0.0001
	Total	4,292,425	77,310	5,756	98	1.64	1.70	0.95	0.7703
West	7	265,867	2,511	1,250	39	0.94	3.12	3.30	< 0.0001
	8	356,656	5,348	5,279	562	1.50	10.65	7.83	< 0.0001
	9	403,559	9,615	13,720	1,368	2.38	9.97	4.54	< 0.0001
	10	547,094	10,394	11,759	568	1.90	4.83	2.62	< 0.0001
	20	394,784	7,054	16,147	1,908	1.79	11.82	7.37	< 0.0001
	Total	1,967,960	34,922	48,155	4,445	1.77	9.23	5.63	< 0.0001
Northwest	11	330,164	3,025	6,350	17	0.92	0.27	0.29	< 0.0001
	12	275,719	2,283	500	1	0.83	0.20	0.24	0.1370
	13	158,969	2,078	3,964	491	1.31	12.39	9.48	< 0.0001
	14	274,862	1,668	3,720	235	0.61	6.32	10.41	< 0.0001
	15	828,229	13,256	8,247	414	1.60	5.02	3.14	< 0.0001
	Total	1,867,943	22,310	22,781	1,158	1.19	5.08	4.26	< 0.0001
North	17	956,008	18,174	10,951	519	1.90	4.74	2.49	< 0.0001
	19	288,438	2,911	1,934	237	1.01	12.25	12.14	< 0.0001
	Total	1,244,446	21,085	12,885	756	1.69	5.87	3.46	< 0.0001

Source: State Worker’s Health Center (Centro Estadual de Saúde do Trabalhador, CEST), 2020.

PR = prevalence ratio; HR = health region.

* Epidemiological Bulletin released on September 30, 2020, available from: https://www.saude.pr.gov.br/sites/default/arquivos_restritos/files/documento/2020-09/informe_epidemiologico_30_09_2020_.pdf

Slaughterhouses represent a significant number of workplaces in Paraná, employing a total of 89,577 workers. The data show a significant number of infected workers, 6,457 in total, accounting for 4.29% of the total number of cases reported in the state. The prevalence of cases in the general population is 1.9%, whereas the prevalence in slaughterhouse workers is 7.21%. This difference was statistically associated with a significance level of p < 0.00001 in the chi-square test (independence test) and a prevalence ratio (PR) of 4.29 (Table 1). This means that slaughterhouse workers have a 4.29-fold higher likelihood of being infected by COVID-19 in comparison with the general population.

Regarding the impact of sick slaughterhouse workers on the region where they work, data for the study period show that in the Western region of Paraná (20th health region), where there are 48,155 people working in slaughterhouses, more than 27% of COVID-19 cases were reported among these workers. In the Northwest region (13th health region), there are 3,964 slaughterhouse workers and approximately 24% of COVID-19 cases in this region were reported among them.

The prevalence of COVID-19 in slaughterhouse workers in the 5th and 14th health regions was below the state average, although there was an increased PR (prevalence of 4.48% and 6.32% and PR of 10.47 and 11.04, respectively). Only the 3rd and 12th health regions were not statistically significant (0.71 and 0.2, respectively). In the 11th health region, COVID-19 infections were more prevalent in the general population than in slaughterhouse workers (p < 0.0001). The 8th, 13th, 19th, and 20th health regions had the highest COVID-19 prevalence and were statistically significant (p < 0.00001).

The PR of COVID-19 among slaughterhouse workers differed between the health regions and macroregions of Paraná. The 19th, 13th, 14th, and 5th health regions had the highest PR rates (13.97, 11.04, 10.67, and 10.47, respectively) and were statistically significant (p < 0.0001). As for the macroregions, the West and the Northwest had the highest PR rates (5.63 and 5.36, respectively) and were statistically significant (p < 0,00001).

## Discussion

The production process in slaughterhouses employs a significant number of workers in the southern region of Brazil. A total of 168,119 jobs are linked to 340 projects in poultry and swine slaughter alone, accounting for more than 60% of the total jobs generated by the sector in the country. These jobs and the regional distribution of municipalities involved in food production are centered in the North-Northwest regions of the state of Rio Grande do Sul, the West of the state of Santa Catarina, and the West and North of the state of Paraná. There are thousands of families and workers directly involved in this production chain, as well as in auxiliary activities, to meet slaughterhouse demands, resulting in a major focus of regional COVID-19 spread.^5^

Study results identified that slaughterhouses are places of significant transmission of COVID-19 among employees. This finding is not random, but rather due to the living and working conditions that these workers are exposed to, which are significantly different from those in the general population and should be further investigated by other studies.

The specific conditions these workers are exposed to in the workplace include large concentrations of workers in closed environments, poor ventilation, low temperatures, humidity, and workstations that do not respect to the minimum safety distance. Public transportation, cafeterias, break rooms, and locker rooms are places where they cannot comply with distancing recommendations. In addition, many of these workers are migrants exposed to situations of social vulnerability who cannot easily access social and health services, which influences further exposure to COVID-19, unlike other groups.^8^

One of the main mechanisms to contain an epidemic is the establishment of specific surveillance strategies that aim to detect the greatest possible number of cases and contacts and the subsequent adoption of actions that reduce the risk of transmission, especially for airborne viruses such as SARS-CoV-2.^9^ In this sense, containing the spread of COVID-19, which happens quickly and manifests itself similarly to other flu-like syndromes, has been one of the main challenges during the pandemic. Thus, health services were put in the spotlight, given that COVID-19 is a community disease that requires a well-prepared health care system.

Since May 2020, the Brazilian Health Surveillance Department has been conducting inspections and guidance in Paraná, requiring slaughterhouses to comply with the preventive measures recommended by the State Department of Health and the Brazilian Ministry of Health. These measures include: a working shift scale to avoid agglomerations and reduce the flow, contact, and number of workers per shift; a distance of at least 1.5 meters between workers in workstations and other shared environments; changes in entry and exit times, access to locker rooms, meal times, and thermal adaptation and psychophysiological breaks to avoid contact, peak hours, and agglomerations, ensuring that workers keep a distance of at least 1.5 meters; provision of hand sanitizer for disinfection; and prohibiting workers from using their co-workers’ equipment or sharing equipment.^10^

After Resolution no. 855 took effect in the state of Paraná, the slaughterhouse industry issued a statement against the recommended distances, claiming that 1.5 meters would result in a 43% drop in production levels and that 1 meter had already resulted in a 18% drop. A few weeks later, the resolution was revoked by the Health Secretary of the state of Paraná.^11^

The issue regarding Resolution no. 855 emphasizes a fundamental conflict in society in which health measures face interests related to economic factors. During a pandemic, safety measures focused on the population should be a priority, given that health plays a role in the country’s social and economic development. Of note, agribusiness remained operational during the pandemic, exposing workers to unsafe working conditions, which reinforces the need to discuss food security in Brazil.

Those who were able to social distance developed, in addition to fear of contracting the virus, concerns about the need to leave the house, changes in routine and sleep patterns, and feelings of sadness or concern.^12^ However, for workers such as those involved in food production, social distancing was not a viable option, both socially and economically, which increased the risks and fears experienced by this significant part of the population and their families.

In other countries, daily activities were interrupted due to the need for social distancing in an effort to stop the spread of COVID-19, which reached 190 countries in less than 4 months, including Brazil. Italy, Spain, and Portugal developed initiatives to avoid agglomerations, which affected the food chain. In these countries, supermarkets implemented rules for accessing and purchasing products as a strategy to avoid shortages.^13^

The relationship between work and COVID-19, although little explored, becomes clear when we recognize that both in Brazil and in China, the first deaths from COVID-19 were of workers infected in their workplace. In Wuhan, the first deaths were of people who worked at the city’s seafood market, which is considered the initial focus of contamination due to the handling of live animals. In Brazil, one of the first victims was a domestic worker who was infected after being exposed to the virus by her employers, who had been in Italy in the beginning of 2019.^14^

It should be noted, however, that health professionals around the world, including those involved in occupational health, have faced several challenges during the pandemic, with the shortage of personal protective equipment (PPE) being the most significant one.^14^ However, the lack of important information to help elucidate the relationship between work, especially in the food production sector, and the contamination and spread of COVID-19 is challenging. There are reports of workers without PPE in food marketing environments because managers have not been able to adapt working conditions to the new routine imposed by COVID-19 and because of a complete lack of knowledge of basic safety measures, agglomerations, and excessive product purchase. These situations could collapse the entire structure that has been put into place to contain the virus and result in early food shortage.^13^

Still regarding risks, rural workers directly involved in food production face significant risks of exposure to the virus because of the contact with different people involved in the production chain, such as transportation and food marketing workers and the worker’s own family, which demonstrates the need to discuss exposure risks throughout the work process. It is worth mentioning that these questions promote new possibilities to discuss agricultural work processes, which are directly involved in food security, as well as consumption patterns and occupational safety during the pandemic.

Of note, based on the discussion of the data and considering the Brazilian context, the troubled way the country coped with the pandemic hindered virus containment. Recommendations such as social distancing and the use of masks were poorly received by the population for many reasons, such as a lack of standardization between recommendations in the country due to political/ideological differences, and the nomination of different health ministers that imposed conflicting sanitary measures during the pandemic, among others.

This fragmentation in the health system was attenuated by the historical decentralized organization of the Brazilian Unified Health System (Sistema Único de Saúde, SUS), which allowed managers and municipal health teams to manage health services with autonomy in relation to the federal government. Thus, the fragmentation and weakening of important channels that allowed for dialogue about food and nutrition security, such as National Council for Food Security, which was recently terminated, demonstrate how challenging the path we are on can be. Food insecurity, which was already a reality, could be exacerbated by the COVID-19 pandemic.^15^

Thus, measures that grant more than 70% of rural production financing to peasant agriculture, which aims at the production of commodities, should be reviewed and redirected to family agriculture, which is better distributed geographically (allows for local supply), produces diversified foods, employs more than 80% of rural workers, and uses more sustainable production practices.^16^ It is worth noting that the guidance, inspection, and preventive measures conducted by Health Surveillance, Epidemiological Surveillance, and Occupational Health Surveillance agencies are essential and reinforce the importance of implementing public policies in the health system.

Alternative food systems have become popular during the pandemic, as they can optimize access to fresh and healthy food, positively impact the economy of some important regions in Paraná, and strengthen the local economy, employing small family farmers in different regions. This reinforces the need for data regarding the exposure risk faced by food industry workers and those who produce food, so that they can gain greater visibility and achieve conditions that are similar to those faced by the rest of the population.

Food security and worker’s health are part of SUS, which is considered the largest public health system in the world and has faced different challenges throughout its history. Of note, even though SUS has been underfunded since its creation, it has been providing the necessary basic support to combat COVID-19, including services, equipment, and an indispensable workforce throughout the national territory. However, the lack of investment, its dismantling, and its disruption become more evident in times of crisis, revealing a lack of resources in the face of current demand.^14^

## Conclusions

Based on the results, we can conclude that certain production sectors, such as slaughterhouses, are highly likely to further spread COVID-19 in the regions where they are located. In addition, they show an important relationship between worker’s health and food security, as these sectors employ a significant number of workers that influence food supply to different regions and countries.

The number of cases of infected workers is significant, 6,457 in total, accounting for 4.29% of the total number of cases reported in the state of Paraná. The prevalence of COVID-19 cases among the general population is 1.9%, whereas the prevalence among slaughterhouse workers is 7.21%. Thus, slaughterhouse workers are more likely to be contaminated by COVID-19, which means that changes in the work environment aiming at improving the safety of these workers should be implemented.

Individual protective measures, although important, do not seem to be sufficient to prevent workers from being exposed to COVID-19. Comprehensive response strategies via social policies for the implementation of interventions that go beyond individual measures that guide “appropriate self-case behaviors” are lacking. For these recommendations to be followed, structural and social changes must occur at all levels of the production sector, including farmworkers, food companies (such as slaughterhouses), and related services, such as public transportation, food marketing, delivery workers, and all those whose occupational activity represents a risk factor for infection.

The government has a crucial role in mitigating the effects of the pandemic, using short-, medium- and long-term measures not only to control the spread of COVID-19, but also to deal with the social, economic, and health consequences. We believe this situation requires constant learning, as it is a new experience that must face the social inequalities that were already present in the whole country.

This study did not aim to negatively expose the slaughterhouses, but rather analyze an important section of occupational health that involves the food chain and its workforce. These workers should be valued and have their safety guaranteed, as they are essential for food security in the country.
